# Role of Vγ9vδ2 T lymphocytes in infectious diseases

**DOI:** 10.3389/fimmu.2022.928441

**Published:** 2022-07-18

**Authors:** Laetitia Gay, Soraya Mezouar, Carla Cano, Paul Frohna, Loui Madakamutil, Jean-Louis Mège, Daniel Olive

**Affiliations:** ^1^ Aix-Marseille Univ, Intitut Recherche pour le Développement (IRT), Assistance Publique Hôpitaux de Marseille (APHM), Microbe, Evolution, Phylogeny, Infection (MEPHI), Marseille, France; ^2^ Immunology Department, IHU-Méditerranée Infection, Marseille, France; ^3^ ImCheck Therapeutics, Marseille, France; ^4^ Aix-Marseille Univ, APHM, Hôpital de la Conception, Laboratoire d’Immunologie, Marseille, France; ^5^ Centre pour la Recherche sur le Cancer de Marseille (CRCM), Inserm UMR1068, Centre national de la recherche scientifique (CNRS) UMR7258, Institut Paoli Calmettes, Marseille, France

**Keywords:** Vγ9Vδ2 T cell, antimicrobial immunity, infectious diseases, butyrophilin, therapeutic approaches

## Abstract

The T cell receptor Vγ9Vδ2 T cells bridge innate and adaptive antimicrobial immunity in primates. These Vγ9Vδ2 T cells respond to phosphoantigens (pAgs) present in microbial or eukaryotic cells in a butyrophilin 3A1 (BTN3) and butyrophilin 2A1 (BTN2A1) dependent manner. In humans, the rapid expansion of circulating Vγ9Vδ2 T lymphocytes during several infections as well as their localization at the site of active disease demonstrates their important role in the immune response to infection. However, Vγ9Vδ2 T cell deficiencies have been observed in some infectious diseases such as active tuberculosis and chronic viral infections. In this review, we are providing an overview of the mechanisms of Vγ9Vδ2 T cell-mediated antimicrobial immunity. These cells kill infected cells mainly by releasing lytic mediators and pro-inflammatory cytokines and inducing target cell apoptosis. In addition, the release of chemokines and cytokines allows the recruitment and activation of immune cells, promoting the initiation of the adaptive immune response. Finaly, we also describe potential new therapeutic tools of Vγ9Vδ2 T cell-based immunotherapy that could be applied to emerging infections.

## Introduction

Gamma-delta (γδ) T cells are « unconventional » T lymphocytes that do not require major histocompatibility complex (MHC) presentation of antigen (1). Human γδ T cells are classified into two main subsets according to the expression of T cell receptor (TCR) δ chain (2). Vδ1 T cells are more common in mucosal tissues and are involved in the first line of the immune defense against solid tumors and infections. The Vδ2 T cells, that is a subset uniquely associated with Vγ9 chain (called Vγ9Vδ2), are abundant in the peripheral blood and play a role of immune effector in tumor surveillance and also in antimicrobial defense (2). Indeed, Vγ9Vδ2 T cells can directly kill infected cells through different mechanisms, and also prime and modulate functions of other innate and adaptive immune cells *via* cytokines, antigen presentation and cell contact to develop antimicrobial immunity (3).

Human Vγ9Vδ2 T cells, typically represent 2 to 5% of peripheral blood T cells, are expanded following infection with a wide range of microbial agents and can represent up to 50% of the peripheral T cell pool (3, 4). This subset of T cells is enriched in the circulation of patients with bacterial infections, including mycobacterial diseases, listeriosis, salmonellosis, brucellosis, tularemia, legionellosis and Q fever (5–11), and with protozoal parasite infections such as malaria, toxoplasmosis and leishmaniasis (12–14). Vγ9Vδ2 T cells are also increased in the bronchoalveolar lavage fluid of patients with active pulmonary tuberculosis or psittacosis (15), and in cerebral spinal fluid from patients with bacterial meningitis (*M. tuberculosis*, *H. influenzae*, *S. pneumoniae*, and *N. meningitidis*); such pattern is corrected by successful antibacterial therapy (16, 17). Bacterial vaginosis is also associated with an increase of Vγ9Vδ2 T cells in the female reproductive tract in women (18, 19). Furthermore, in patients with *P. falciparum* malaria, an increase in Vγ9Vδ2 T lymphocytes in human spleens during infection has also been observed (20). Globally, the rapid expansion of circulating Vγ9Vδ2 T lymphocytes during acute infections as well as their localization at the site of active disease indicate that Vγ9Vδ2 T cells may play an important role in the immune response to infection.

In contrast, it seems that the number of Vγ9Vδ2 T cells in the blood is reduced in patients with a viral infection (21–24). In patients with chronic hepatitis B, the frequency of peripheral and hepatic Vγ9Vδ2 T cells decreases with disease progression. Similarly, the frequency of Vγ9Vδ2 T cells is markedly reduced in the blood and the mucosal tissues of HIV patients, and interestingly is restored with highly active antiretroviral therapy (HAART) (25–27). These observations indicate that Vγ9Vδ2 T cells are activated early after infection but are lost if infection is not controlled. Recently, a decrease in the number of circulating Vγ9Vδ2 T cells has been reported in patients with coronavirus, especially SARS-CoV-2, which was followed by a return to normal levels in recovered patients (22, 23). The aim is to review the mechanisms of Vγ9Vδ2 T cell-mediated antimicrobial immunity and to report the potential therapeutic application of Vγ9Vδ2 T cell immunotherapy to infectious diseases.

## Vγ9vδ2 T cell recruitment to the site of inflammation and their implication in tissue repair

**Figure 1:**
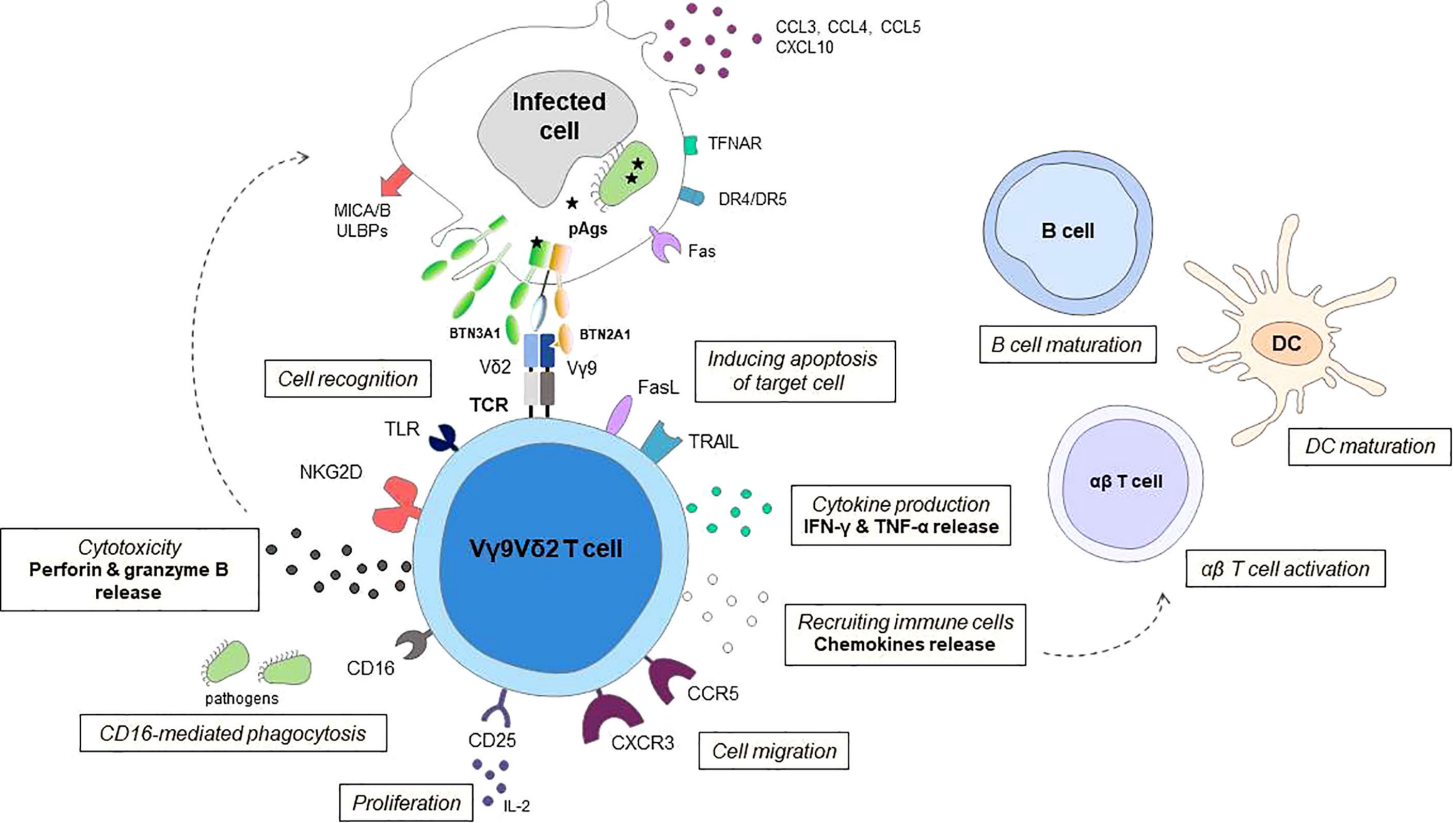
Schematic representation of effector mechanisms of Vγ9Vδ2 T cells in response to infection Vγ9Vδ2 T cells can distinguish between infected cells and normal cells using T cell receptor (TCR) and other cellular receptors especially natural killer group 2 member D receptor the (NKG2D) to sense isopentenyl pyrophosphate (IPP) levels and stress signals (such as MICA/B, ULBPs) displayed on target cells. The butyrophilin receptors BTN3A1 and BTN2A1 on target cells act to detect (pAgs) such as HMBPP and as a direct ligand for the Vγ9Vδ2 T cell receptor. Human Vγ9Vδ2 T cells can also recognize danger signals directly from pathogens through Toll-like receptors (TLRs). Following activation, Vγ9Vδ2 T cells kill infected cells by releasing lytic mediators (perforin, granzyme B), and pro-inflammatory cytokines, inducing target cell apoptosis *via* Fas/FasL, TNF-related apoptosis-inducing ligand (TRAIL) and TNF-α pathways, and antibody-dependent cell-mediated cytotoxicity (ADCC) through CD16 expression. In a CD16-dependent manner, Vγ9Vδ2 T cells may also have phagocytic functions. The chemokine receptors, including CCR5, control the ability of Vγ9Vδ2 T cell to migrate to the site of infection. The release of chemokines and cytokines allows recruitment of immune cells, enhance antigen priming of dendritic cells (DCs) and maturation of B cells. Vγ9Vδ2 T cells can display an APC-like phenotype and are able to present Ags and provide costimulatory signals sufficient for strong induction of αβ T cells, promoting the initiation of the adaptive immune response. The survival and proliferation of Vγ9Vδ2 T cells are mostly modulated by different cytokines, such as IL-2.

The traffic of leukocytes to tissues is an essential step for the development of an immune response that is mainly controlled by the interactions between chemokines and their specific receptors (28). During infection the onset of local inflammation is associated with an increased chemokine production that plays a role in transendothelial migration of Vγ9Vδ2 T cells into the tissues. The majority of circulating Vγ9Vδ2 T cells have the potential to be rapidly recruited in tissues during the course of infection, due to their expression of inflammatory homing chemokine receptor CCR5 and CXCR3 (**Figure 1**) (28, 29). Indeed, CCR5 expressed on activated Vγ9Vδ2 T cells mediates their migration to influenza virus-infected sites (30). Similarly, high levels of CCR5 and CXCR3 receptors on Vγ9Vδ2 T cells are responsible of transendothelial migration of cells to the lungs in monkeys infected with *M. tuberculosis* or Bacille Calmette-Guerin (BCG) (31). A macaque model showed that Vγ9Vδ2 T cells exhibit trans-endothelial migration, interstitial localization, and granuloma infiltration in response to *M. tuberculosis* infections (32).

In addition to be anti-microbial effectors, Vγ9Vδ2 T cells, once activated locally or recruited to tissue compartments, might also participate to tissue repair or wound healing after post-infectious tissue damage. In acute bacterial peritonitis, Vγ9Vδ2 T cells accumulate rapidly at the site of infection and likely contribute to scarring in the peritoneal cavity, both directly *via* the local release of IFN-γ, and indirectly *via* induction of IL-6 production by mesothelial cells and peritoneal fibroblasts (33, 34). In addition, migrating Vγ9Vδ2 T cells can locally produce fibroblast growth factor-7 (FGF-7), a homeostatic mediator against tissue damages induced by bacterial infections (35). In a macaque model, induced expansion of Vγ9Vδ2 T cells by treatment with 4-hydroxy-3-methyl-but-2-enyl pyrophosphate (HMBPP) and IL-2 led to the apparent attenuation of plague lesions in lungs (35). These Vγ9Vδ2 T cells may therefore contribute to immune responses or tissue homeostasis against bacterial infections.

## Recognition of infected cells by Vγ9vδ2 T cells

### Recognition by phosphoantigens

In humans, Vγ9Vδ2 T cells recognise small pyrophosphate-containing molecules called phosphoantigens (pAgs) present in the malignant target cell or in the infected cells (**Figure 1**) (29). These small molecules are isopentenyl pyrophosphate (IPP) produced by infected cells or HMBPP produced by certain bacteria (*Mycobacterium tuberculosis*, *Listeria monocytogenes*) and parasites (*Plasmodium falciparum*, *Toxoplasma gondii*). It is important to note that the naturally occurring pAg HMBPP stimulates Vγ9Vδ2 T cells about 10,000-fold more efficiently than IPP (15, 36, 37), this recognition provides a formal basis for the role of Vγ9Vδ2 T cells in anti-infective immunity (38–40). A recent study showed that Vγ9Vδ2 T cell activation can occur independently of HMBPP produced by the bacteria but *via* the regulation of host cholesterol biosynthesis (41). Indeed, infection of human dendritic cells (DCs) with HMBPP-negative *L. monocytogenes* results in an upregulation of cholesterol metabolism in these cells, leading to increased intracellular IPP levels and direct activation of Vγ9Vδ2 T cells. On the other hand, Vγ9Vδ2 T cells can recognize a mycobacterial glycolipid component, 6-O-methylglucose lipopolysaccharide, which promotes TCR-dependent effector functions of Vγ9Vδ2 T cells against *M. tuberculosis in vitro* (42).

The recognition mechanisms of pAgs by Vγ9Vδ2 T cells involve the butyrophilin (BTN) protein family. The butyrophilin 3A1 (BTN3A1, CD277), expressed by both immune cells and somatic cells (43), directly binds pAg intracellularly through its B30.2 cytoplasmic domain leading to a conformational change in its ectodomain that is sensed by Vγ9Vδ2 T cells (44–46). BTN3A1 interacts at the plasma membrane with another member of the BTN family, BTN2A1 which is a direct ligand for the Vγ9 TCR chain, thus ensuring the synapse between Vγ9Vδ2 T cells and target cells (47–49). Several studies have confirmed that Vγ9Vδ2 T cell activation is dependent on BTN3A during infections. Indeed, the BTN3A blocking antibody (103.2 mAb) was able to inhibit the degranulation of Vγ9Vδ2 T cells when they were co-cultured with cells infected with *M. tuberculosis*, *L. monocytogenes*, *P. falciparum* or Epstein-Barr virus (38, 41, 42, 44, 50).

The expression of these butyrophilins can be modulated by infection in some cases. Indeed, the plasma membrane expression of BTN3A and BTN2A was induced on *P. falciparum* infected red blood cells (iRBCs) (38). In addition, we recently showed that intracellular bacteria, *M. tuberculosis* and *C. burnetii* increased BTN3A and BTN2A expression on monocytes, concomitantly to Vγ9Vδ2 T cell activation (manuscript submitted). In contrast, human immunodeficiency virus (HIV) infection did not appear to enhance BTN3A expression on DCs (51), indicating that basal BTN3A expression maybe sufficient for translating pAgs signal in HIV-infected cells.

### Recognition *via* Nkg2d (natural killer group 2 member D) receptor

Other transmembrane activatory receptors, notably the NKG2D receptor, have been implicated in the effective triggering of antimicrobial responses by Vγ9Vδ2 T cells. Indeed, NKG2D can bind to its ligands including MICA/B (MHC class I-related chain proteins A and B) and UL16-binding proteins (ULBP1-4). Besides their expression on tumor cells, these ligands are upregulated on cells infected by Zika virus and EBV (52–55). This is also the case during infection with intracellular bacteria, for instance MICA is upregulated by DCs infected with *M. tuberculosis* (56) and ULBP1 by macrophages infected with *M. tuberculosis* and *Brucella* (57, 58).

### Recognition *via* toll-like receptors

Human γδ T cells also recognise danger signals from pathogens *via* TLRs. Vγ9Vδ2 T cells can be activated by TLR3 and TLR4 ligands and exhibit enhanced antibacterial responses (59). On the other hand, TLR8 ligands were shown to inhibit the expansion of Vγ9Vδ2 T cells *in vitro*, while these can be potent co-stimuli for Vγ9Vδ2 T cell activation in a monocyte-dependent manner (60). Hence, Vγ9Vδ2 T cells may recognize infected cells through several different receptors involved in innate immune responses.

## Antimicrobial responses of vγ9vδ2 T cells

### Vγ9vδ2 T cells Kill infected cells in an innate immune manner

Human Vγ9Vδ2 T cells exert both a direct cytotoxic activity against pathogen-infected cells as well as a cell-mediated non-cytolytic activity based on cytokine production (**Figure 1**) (**Table 1**). Regarding direct cytotoxicity, Vγ9Vδ2 T cells have been shown to kill cells infected by *M. tuberculosis*, *Brucella suis*, *Listeria monocytogenes*, *P. falciparum* and influenza virus *in vitro*, through the secretion of cytolytic molecules such granzymes, granulysin and perforin (64–66, 68, 71, 74, 75), similar to their responses to malignat cells. In addition, apoptosis triggered by death inducible receptors, including Fas and tumor necrosis factor-related apoptosis-inducing ligand receptors (TRAIL), is a major mechanism of Vγ9Vδ2 T cells involved in the elimination of cells infected by Epstein-Barr and influenza virus (78, 79, 84, 93). Furthermore, engagement of NKG2D is sufficient to induce cytokine production and release of lytic granules; it increases TCR-dependent effector functions of Vγ9Vδ2 T cells in *M. tuberculosis* and *Brucella* infections (56, 58). In contrast, in other studies on *M. tuberculosis* or *L. monocytogenes*, NKG2D was not involved (41, 94). These discrepancies may be due to the different expression of NKG2D ligands between infections and between cell populations. On the other hand, NKG2D activation is required for Vγ9Vδ2 T cell cytotoxicity in viral infections with Epstein-Barr, influenza and Zika viruses (82).

**Table 1:** Summary table of the main involvement of Vγ9Vδ2 T cells in infectious diseases.

	Infections	Human Vγ9Vδ2 T cells	Mechanisms of antimicrobial immunity	Vγ9Vδ2 T cell “memory” responses
**Bacteria**	*M. tuberculosis*	↑ in blood, bronchoalveolar lavage fluid and cerebral spinal fluid (5, 15, 16) ↓ loss of cytotoxic activity (61–63)	- IFN-γ, TNF-α, perforin, granzymes, and granulysin release (64–66) - NKG2D activation (58)	BCG vaccination: recall expansion in humans and in macaques (67)
	*L. monocytogenes*	↑ in blood (6)	- IFN-γ, TNF-α, IL-4, IL-17, and perforin release (68)	*L. monocytogenes* secondary infection: recall expansion in macaques and in mice (69, 70)
	*Brucella* spp.	↑ in blood (8)	- IFN-γ and perforin release (71) - Fas-mediated signals (71) - NKG2D activation (58)	Restore the full functional capacity of *Brucella*-infected DCs (72)
**Parasite**	*P. falciparum*	↑ in blood and spleens (20) ↓ loss of cytotoxic activity (12, 73)	- IFN-γ, granzymes and granulysin release (74, 75) - phagocytosis (38)	*P. falciparum* sporozoite vaccine: recall expansion associated with protection in humans (76, 77)
**Virus**	Influenza	Not known	- IFN-γ, perforin and granzymes release (78, 79) - TRAIL and Fas-mediated signals (78, 79) - NKG2D activation (78, 79)	- Help to produce influenza virus-specific Ab (80, 81) - Influenza vaccination: memory responses (82, 83)
	SARS-CoV	↑ in blood after clearing SARS-CoV-1 and SARS-CoV-2 infections (21–23)	- IFN-γ release (21)	Correlation with higher anti-SARS-CoV-1 specific IgG titers (21)
	Epstein-Barr	↑ in blood (50)	- TRAIL and Fas mediated signals (84) - NKG2D activation (50, 84)	Not known
	HBV/HCV	↓ in blood in chronic hepatitis (24) inability of cytotoxic activity (85–87)	- IFN-γ release (88)	Not known
	HIV	↓ in blood and mucosal tissues inability of cytotoxic activity (25–27, 61)	- ADCC mediated cytotoxicity (89) - production of antiviral factors that block HIV replication *in vitro* (90)	- DC maturation and HIV-specific CD8^+^ T cell responses (91) - HIV Env-specific Ab titers during chronic SHIV (92)

The arrow ↑ represents an increase and the arrow ↓ indicates a decrease in the number of Vγ9Vδ2 T cells.

Vγ9Vδ2 T cells have also been shown to be able of antibody dependent cell-mediated cytotoxicity (ADCC). Indeed, upon stimulation by pAgs, Vγ9Vδ2 T cells express CD16 (FcγRIIIa), an activatory Fcγ receptor that is constitutively expressed on NK cells and mediates ADCC (95, 96). Although total numbers of Vγ9Vδ2 T cells are decreased during HIV infection, resilient activated CD16+ Vγ9Vδ2 T cells were shown to retain the ability to induce ADCC and exert their antiviral functions in HIV disease (89). Moreover, Vγ9Vδ2 T cell expression of CD16 is increased in children in malaria-endemic regions, suggesting a potential role for Vγ9Vδ2 T cells in inciting antibody-mediated parasite killing (97). Besides ADCC, a recent study showed that Vγ9Vδ2 T cells destroy *P. falciparum* infected red blood cells (iRBCs) by a CD16-dependent phagocytosis mechanism (38). As a matter of fact, there are data suggesting that Vγ9Vδ2 T cells can phagocytose particles and act as professional antigen-presenting cells (pAPCs). In response to *E. coli*, peripheral human Vγ9Vδ2 T cells transitioned from cytokine-producing bacterial effectors to professional phagocytic killers in a CD16-dependent manner (98, 99). A recent study also showed that Vγ9Vδ2 T cells suppress *P. falciparum* by direct killing and phagocytosis (38).

Regarding cell-mediated non-cytolytic activity, there are abundant data documenting the pivotal role of IFN-γ and TNF-α secretion on Vγ9Vδ2 T cell responses during infection. In Vγ9Vδ2 T cell-depleted humanized mice, decreased resistance to acute lethal infections with *Staphylococcus aureus*, *Escherichia coli*, and *Morganella morganii* correlated with decreased serum IFN-γ titers, a cytokine known to control numerous bacterial infections (100). The release of IFN-γ is part of the effector mechanisms of Vγ9Vδ2 T cells in *M. tuberculosis, B. suis* and *P. falciparum* infection (64, 71, 101), and also inhibits influenza virus, HCV and SARS-CoV-1 replication (21, 30, 88, 102). Early in HSV-induced inflammation, activated Vγ9Vδ2 T cells secrete IFN-γ and TNF-α, and chemokines, that may affect the course of inflammation (19). Finally, the production of chemokines MIP-1α, MIP-1β and RANTES by Vγ9Vδ2 T cells has also been shown to block HIV replication *in vitro* by inhibiting the CCR5 co-receptor that is required for HIV entry (90).

### Vγ9vδ2 T cells cooperation with immune cells

Vγ9Vδ2 T cells contribute to responses against pathogen infection by modulating indirectly the function of other immune cells. Activated Vγ9Vδ2 T cells can induce recruitment of immune cells by secreting chemokines and stimulating monocytes, neutrophils, DCs, B lymphocytes, and different subtypes of T cells through cytokine secretion, notably IFN-γ (**Figure 1**) (103–106). In patients with acute bacterial peritonitis, Vγ9Vδ2 T cells that accumulate at the site of infection favor the recruitment of monocytes, neutrophils, and lymphocytes and produce inflammatory cytokines that are controlled by BTN3A, as demonstrated by the inhibitory effect of BTN3A antagonist mAb 103.2 in this process (33). Vγ9Vδ2 T cells may impact DC function during infection. Indeed, Vγ9Vδ2 T cells may enhance DC activation through IFN-γ secretion and CD4^+^ cell responses to *S. aureus* (106). Several intracellular bacterial pathogens including *M. tuberculosis*, *B. suis*, *C. burnetii*, interfere with DC maturation, which results in poor priming of the adaptive immune response (107, 108). *Brucella*-infected DCs trigger Vγ9Vδ2 T cells activation that required cell-to-cell contact. In turn, co-culture with activated Vγ9Vδ2 T cells resulted in maturation of *Brucella*-infected DCs with increased expression of co-stimulatory CD80 and CD86, and enhanced IFN-γ and IL-12 secretion (72). In ten HIV patients naive of antiretroviral therapy, treatment with zoledronate and recombinant IL-2 achieved not only Vγ9Vδ2 T cells expansion and activation but also DC maturation and HIV-specific CD8^+^ T cell responses, although the eventual interaction between these immune compartments was not explored in the study (91).

Vγ9Vδ2 T cells were shown to induce differentiation and migration of neutrophils through the production of IL-17 during *M. tuberculosis*, *L. monocytogenes* infections and in bacterial meningitis (17, 109). Moreover, Vγ9Vδ2 T cells respond rapidly to neutrophils after phagocytosis of a broad range of bacteria at the site of infection, and in turn mediate the local differentiation of neighbouring neutrophils into APCs for both CD4^+^ and CD8^+^ T cells *in vitro* (110).

Vγ9Vδ2 T cells can also promote adaptive-like responses by sharing functions with APCs (111). Indeed, Vγ9Vδ2 T cells promote efficient adaptive immunity through processing and presenting influenza virus-derived peptides to CD4^+^ and CD8^+^ T cells (80, 81). In malaria patients, Vγ9Vδ2 T cells presented increased plasma membrane expression of APC markers HLA-DR and CD86. Similarly, in response to infected red blood cells *in vitro*, Vγ9Vδ2 T cells show an APC-like phenotype and are able of Ag presentation and αβT cell activation *in vitro* (112). Vγ9Vδ2 T cells may therefore promote the initiation of the adaptive response despite a possible impairment of conventional APCs. In response to *E. coli* and *L. monocytogenes*, human Vγ9Vδ2 T cells also display APC functions (99, 113). Futhermore, phosphoantigen-activated Vγ9Vδ2 T cells can inhibit IL-2-induced expansion of Tregs and reverse subsequent suppression of mycobacterium-specific T-cell immune responses (114).

Finally, it is well known that γδ T cells have a strong impact on humoral immunity. A subset of human Vγ9Vδ2 T cells isolated from peripheral blood expresses the CXC chemokine receptor type 5 (CXCR5) like T follicular helper cells, and, upon antigen stimulation, they are able to express the costimulatory molecules ICOS and CD40L, to produce cytokines such as IL-2, IL-4, and IL-10, and to help B cells for antibody production (**Figure 1**) (115, 116). In addition, Vγ9Vδ2 T cells activated with the phosphoantigen HMBPP and in presence of IL-21 can also influence the localization of B cell inside the germinal center, positioning them into the light zone thanks to the production of CXC motif chemokine 13 (CXCL13) (116). Surprisingly, during chronic Simian-Human Immunodeficiency Virus (SHIV) infection, Vγ9Vδ2 T cell activation boosted HIV Env-specific Ab titres (92). It has also been reported that human Vγ9Vδ2 T cells facilitated H9N2 influenza virus specific IgG production (81), and that the higher number of circulating Vγ9Vδ2 T cells was associated with higher anti-SARS-CoV-1 specific IgG titers (21).

### Vγ9vδ2 T cell “memory” responses

The Vγ9Vδ2 T cells may acquire a memory effector phenotype (T_EM_ cells) following several infections, as shown by the expression of the memory and activation markers CD27 and CD45RA. This phenotype has been reported in bacterial (31), parasitic (76), and viral infections (21, 22, 117).

In macaques, a clear memory-type response of Vγ9Vδ2 T cells was detected as early as four days after BCG re-infection and the magnitude of this expansion was 2-9-fold greater than that seen during primary BCG infection (67). A recall expansion of Vγ9Vδ2 T cells was also observed in macaques infected with *L. monocytogenes* or challenged with Salmonella and smallpox vaccines (69, 70, 118). In addition, studies in cattle and pigs showed similar responses to those found in macaques with a rapid γδ T cell proliferation after BCG vaccination (119–123). These observations demonstrate the essential role of γδ T cells in developing a long-term immunity against pathogens.

It is difficult to determine in humans whether a Vγ9Vδ2 T cell expansion observed during an infection represents a primary or recall response. Interestingly, Vγ9Vδ2 T cells induced by BCG or influenza vaccination develop memory responses (83, 124), and the numbers of memory Vγ9Vδ2 T cells correlates with protection in an *P. falciparum* sporozoite vaccine trial in a malaria endemic region (77). These data suggest that immunotherapy based on Vγ9Vδ2 T cells, which contribute to adaptive immunity, represents a great potential for the treatment of infections.

Overall, Vγ9Vδ2 T cells may act as an antimicrobial defense through different molecular mechanisms and also constitute a memory cell population that provides protection against subsequent infection. Hence, human Vγ9Vδ2 T cells may affect the progression and outcome of infectious diseases.

## Vγ9vδ2 T cell deficiencies in infectious disease

Alterations of Vγ9Vδ2 T cell phenotype and/or functions have been reported in several infections usually due to intracellular pathogens. Hence, a loss of CD27 expression on circulating Vγ9Vδ2 T cells was reported in patients with active tuberculosis, suggesting an impairment of effector functions (61, 62). Indeed, Vγ9Vδ2 T cell expansion was accompanied by the dramatic reduction of the Vγ9Vδ2 T cells effectors (T_EM_ and T_EMRA_ cells), with decreased IFN-γ production and granulysin expression. This deficiency was restored by successful antimycobacterial therapy. A loss of cytotoxic activity is also observed in lung Vγ9Vδ2 T cells (63). These results suggest that a high bacterial burden leads to chronic stimulation of effector Vγ9Vδ2 T cells that may result in their loss or exhaustion. As a matter of fact, The progressive loss of reactive Vγ9Vδ2 T cells from the blood and bronchoalveolar fluid in pulmonary tuberculosis patients paralleles upregulation of FasL expression on Vγ9Vδ2 T cells resulting in fratricidal killing (1, 125). A progressive attenuation of the Vγ9Vδ2 response was also observed in children with high parasitaemia in malaria (73). Similarly, prophylaxis with antimalarial drug dihydroartemisinin-piperaquine (DHA-P) during early childhood prevents the development of dysfunctional Vγ9Vδ2 T cells (12, 73).

Patients with chronic HBV infection are usually characterized by a population of exhausted T cells, similarly the ability of Vγ9Vδ2 T cells to proliferate and to respond to a chemotactic signal is diminished, which may explain the reduced frequency of Vγ9Vδ2 T cells in the liver of these patients (85). In HIV and chronic HCV patients, peripheral Vγ9Vδ2 T cells are unable to proliferate and specifically to expand the cytotoxic subset (27, 61, 86, 87). In addition, it has been demonstrated that, during HIV infection, myeloid-derived suppressor cells (MDSC) are expanded and their frequency is inversely correlated with the capacity of Vγ9Vδ2 T cells to produce IFN-γ. However, *in vitro* MDSC depletion did not completely restore IFN-γ production by Vγ9Vδ2 T cells from HIV patients (126), suggesting that during HIV infection MDSC are not the unique player in dampening Vγ9Vδ2 T cell response. Finally, in chronic HCV infection and in HIV/HCV co-infection, direct acting antivirals (DAA) fail to restore Vγ9Vδ2-induced IFN-γ production. In contrast to other T cell subsets, Vγ9Vδ2 T cell dysfunction may persist in liver despite a successful HCV treatment for a reason that remains to be elucidated (87).

Overall, these data support a crucial role for Vγ2Vδ2 T cells in infectious diseases, since functional alterations of these cells can have a significant impact on the outcome of the infectious pathology.

## Vγ9vδ2 T cell-based emerging therapeutic approaches

Overall, the data summarized above indicate that triggering Vγ9Vδ2 T cell cytotoxicity may be a promising strategy for the treatment of infectious diseases caused by intracellular pathogens. Specifically, proliferative, cytotoxic, and cytokine responses of human Vγ9Vδ2 T cell subset are induced by bisphosphonates, such as pamidronate (PAM) and zoledronic acid (Zol), through the intracellular accumulation of IPP and its metabolites. The administration of PAM, a common treatment for osteoporosis and Paget’s disease, to humanized mice decreases the disease severity and mortality caused by human influenza virus infection and EBV-induced lymphoproliferative disease by enhancing Vγ9Vδ2 T cells immunity (84, 93). On the other hand, Zol, a treatment for bone disease, is broadly used *in vitro* and *ex vivo* to stimulate effector Vγ9Vδ2 T cells (127). Zoledronate affects HCV, HCMV and West Nile virus replication by expanding IFN-γ-producing Vγ9Vδ2 T cells (88, 128, 129). As mentioned previously, low-dose IL-2 synergizes with bisphosphonates and hence, is an effective method to activate and expand Vγ9Vδ2 T cells both *in vitro* and *in vivo*. In HIV patients, Zol along with IL-2 allowed the rapid expansion of CD16-expressing T Vγ9Vδ2 cells *in vitro*, associated with enhanced ADCC cytotoxicity (130). In macaques, HMBPP/IL-2 administration induced remarkable Vγ9Vδ2 T cell expansion and resulted in apparent attenuation of plague lesions in lung tissues caused by *Yersinia pestis* infection (35). Similarly, Picostim (similar to HMBPP except one carbon difference)/IL-2 administration induced activation and expansion of effector Vγ9Vδ2 T cells during both the acute and chronic phases of SHIV infection and also increased resistance to tuberculosis in macaques (131), supporting a rationale to explore Vγ9Vδ2 T cell-targeting as treatment of drug-resistant tuberculosis or HIV-associated tuberculosis. Furthermore, IL-12 and also IL-15 enhance the proliferation and expansion of HMBPP-activated Vγ9Vδ2 T cells with effector functions capable of inhibiting intracellular mycobacterial growth (108, 132). On the other hand, IL-18 enhances the proliferative, cytotoxic and recall response of Vγ9Vδ2 T cells from HIV-1-infected individuals (133). In HIV seropositive individuals, where Vγ9Vδ2 T cells are typically reduced even after effective antiretroviral therapy and CD4 T-cell reconstitution, therapies directed at restoring the antiviral activity of Vγ9Vδ2 T cells represent an appealing potential treatment. This raises questions about the therapeutic use of these cells, including the minimal requirement for eliciting a response and the cytokines required for the boost of immune response. A new strategy for treating influenza virus infection has been suggested using the combination of PAM and CD137 agonist. Indeed, activation of the CD137/CD137L pathway could maintain the survival of Vγ9Vδ2 T cells, this may provide a new solution to avoid Vγ9Vδ2 T cell exhaustion and to increase the efficacy of γδ T cell-based immunotherapy (134). However, the clinical use of bisphosphonates as an anti-infective agent has certain limitations. Indeed, it has been reported that repeated pAg treatment may lead effector cells to a senescent or exhausted phenotype, and even lead to their death (135). Better antigens should be sought to help stimulating Vγ9Vδ2 T cells *in vitro*.

Besides pAg-induced activation of Vγ9Vδ2 T cells, a recently novel approach involved the development of a new class of molecules called immunoantibiotics, notably the IspH inhibitor, has been described as also inducing the expansion and activation of human Vγ9Vδ2 T cells (136). IspH, an enzyme in the isoprenoid synthesis pathway, is essential for the survival of most Gram-negative bacteria and the lack of IspH causes an accumulation of its substrate HMBPP, thus allowing the activation of cytotoxic Vγ9Vδ2 T cells. In a humanized mice model of *E. coli* infection, these prodrugs resulted in Vγ9Vδ2 T cell expansion and a lower bacterial load in the tissues (136). This strategy synergises direct antibiotic action with rapid immune response. In addition, these prodrugs allow the targeting of existing multi-resistant microbes (136), as well as decrease the chances of resistance emerging. Unlike antibiotics derived from natural sources, no IspH inhibitors have been discovered in microorganisms, which justify their therapeutical use (137).

Another approach would be to target specifically the ligands expressed on the plasma membrane of stressed cells, such as BTN3A, which are responsible for activation and effector functions of Vγ9Vδ2 T cells. Indeed, an important tool generated in BTN3A research are activating mAbs including the anti-BTN3A agonist 20.1, that mimics the pAg-induced Vγ9Vδ2 T cell activation (43, 138, 139). After successfully showing proof-of-concept of preclinical efficacy (140), another BTN3A agonist mAb, ICT01, is currently under evaluation in the EVICTION phase I/II clinical trial (NCT04243499) sponsored by ImCheck Therapeutics in patients with solid tumors and hematological malignancies (141, 142).

The activating anti-BTN3A mAb could represent important therapeutic tools in infections to overcome the imbalances in immune responses observed in some patients. In this context, we are currently testing the ability of the agonist anti-BTN3A 20.1 to modulate viral/bacterial replication *in vitro* in co-cultures of infected cells with Vγ9Vδ2 T cells (143). By enhancing Vγ9Vδ2 T cell cytotoxicity against infected cells, anti-BTN3A agonist antibodies could offer an alternative treatment strategy for infectious diseases. Combinations of newly emerging therapy with established treatments could minimize the potential side effects of immune reconstitution in the future.

## Conclusion and perspectives

The unique features of Vγ9Vδ2 T cells make these cells ideal candidates that could be targeted to induce protective and durable immunity in the context of infectious diseases. Therapies must be developed to enhance the effector functions of these cells at the site of infection, which would be relevant especially in chronic infections such as HIV infection or tuberculosis where the effector Vγ9Vδ2 T cells are impaired. For the preparation of large number of cells for adoptive cell transfer, it is necessary to identify and develop better antigens, which stimulate the Vγ9Vδ2 T cells expansion *in vitro*. Targeting key receptors such as the BTN3A and BTN2A involved in activation and recognition of Vγ9Vδ2 T cells emerge as potential therapeutic strategies in infectious diseases. Therefore, further research might shed more light on the in-depth understanding of the underlying mechanisms of the antigen recognition and key factors influencing the Vγ9Vδ2 T cell activation during infectious diseases, which will be pivotal for developping effective Vγ9Vδ2 T cell-based therapies against pathogen infections.

## Author contributions

LG, SM, and CC wrote/revised the manuscript. J-LM and DO supervised/revised the manuscript. All authors reviewed the manuscript and contributed to the work.

## Funding

LG was supported by a Cifre fellowship from ImCheck Therapeutics.

## Conflict of interest

DO is cofounder and shareholder of Imcheck Therapeutics, Emergence Therapeutics, Alderaan Biotechnology and Stealth. IO. CC, PF and LM are employees and shareholders of Imcheck Therapeutics.

The remaining authors declare that the research was conducted in the absence of any commercial or financial relationships that could be constructed as a potential conflict of interest.

## Publisher’s note

All claims expressed in this article are solely those of the authors and do not necessarily represent those of their affiliated organizations, or those of the publisher, the editors and the reviewers. Any product that may be evaluated in this article, or claim that may be made by its manufacturer, is not guaranteed or endorsed by the publisher.
